# Insights Into Vestibulo-Ocular Reflex Artifacts: A Narrative Review of the Video Head Impulse Test (vHIT)

**DOI:** 10.7759/cureus.55982

**Published:** 2024-03-11

**Authors:** Melissa Castillo-Bustamante, Henrique F Pauna, Rafael da Costa Monsanto, Veronica A Gutierrez, Jorge Madrigal

**Affiliations:** 1 Otoneurology, Centro de Vértigo y Mareo, Mexico City, MEX; 2 Otolaryngology, School of Health Sciences and Medicine, Universidad Pontificia Bolivariana, Medellín, COL; 3 Otolaryngology, Hospital Universitario Cajuru, Curitiba, BRA; 4 Otopathology, University of Minnesota, Minneapolis, USA; 5 Audiology, Interacoustics Mexico, Mexico City, MEX

**Keywords:** electronic devices, vestibulo-ocular reflex, artifacts, video head impulse test, vestibular testing

## Abstract

Video head impulse test (vHIT) artifacts are defined as spurious elements or disturbances in the recorded data that deviate from the true vestibulo-ocular reflex response. These artifacts can arise from various sources, encompassing technological limitations, patient-specific factors, or environmental influences, introducing inaccuracies in vHIT outcomes. The absence of standardized criteria for artifact identification leads to methodological heterogeneity. This narrative review aims to comprehensively examine the challenges posed by artifacts in the vHIT. By surveying existing literature, the review seeks to elucidate the multifaceted nature of artifacts arising from technological, patient-related, evaluator-related, and environmental factors.

## Introduction and background

In the landscape of vestibular diagnostics, the video head impulse test (vHIT) has emerged as an indispensable tool for evaluating the integrity of the vestibulo-ocular reflex (VOR) [1]. As the vHIT gains widespread adoption in clinical practice, addressing the myriad artifacts influencing its outcomes becomes imperative [1,2]. The meticulous recognition and mitigation of artifacts in vestibular diagnostics, specifically within the domain of the vHIT are of paramount importance for maintaining the accuracy and reliability of test outcomes [1-3]. Artifacts, stemming from diverse sources encompassing technological limitations to patient and evaluator-specific variables, possess the potential to introduce unintended influences on vHIT results [4]. The precise identification and understanding of these artifacts are critical for accurate test interpretation, ensuring that clinical decisions and interventions are based on a nuanced comprehension of vestibular function [5]. Furthermore, as the vHIT increasingly assumes a central role in clinical decision-making and research endeavors, the awareness of artifacts becomes indispensable for upholding the validity of study findings and contributing robust data to the scientific milieu [4,5]. Embracing a comprehensive approach to recognizing artifacts not only refines the diagnostic utility of the vHIT but also catalyzes advancements in technology and methodology, thereby fostering continual enhancement in the precision and applicability of the vHIT within the realm of vestibular diagnostics and research [6]. 

Through a narrative review of existing literature, this article not only identifies prevalent pitfalls but also introduces a glance at methodologies and strategies crafted to mitigate the impact of artifacts on vHIT accuracy. The primary aim is to enhance the reliability and accuracy of the vHIT as a diagnostic modality, thereby advancing our comprehension of vestibular function and elevating the standard of patient care in the dynamic field of vestibular medicine.

## Review

Methods

**Figure 1:**
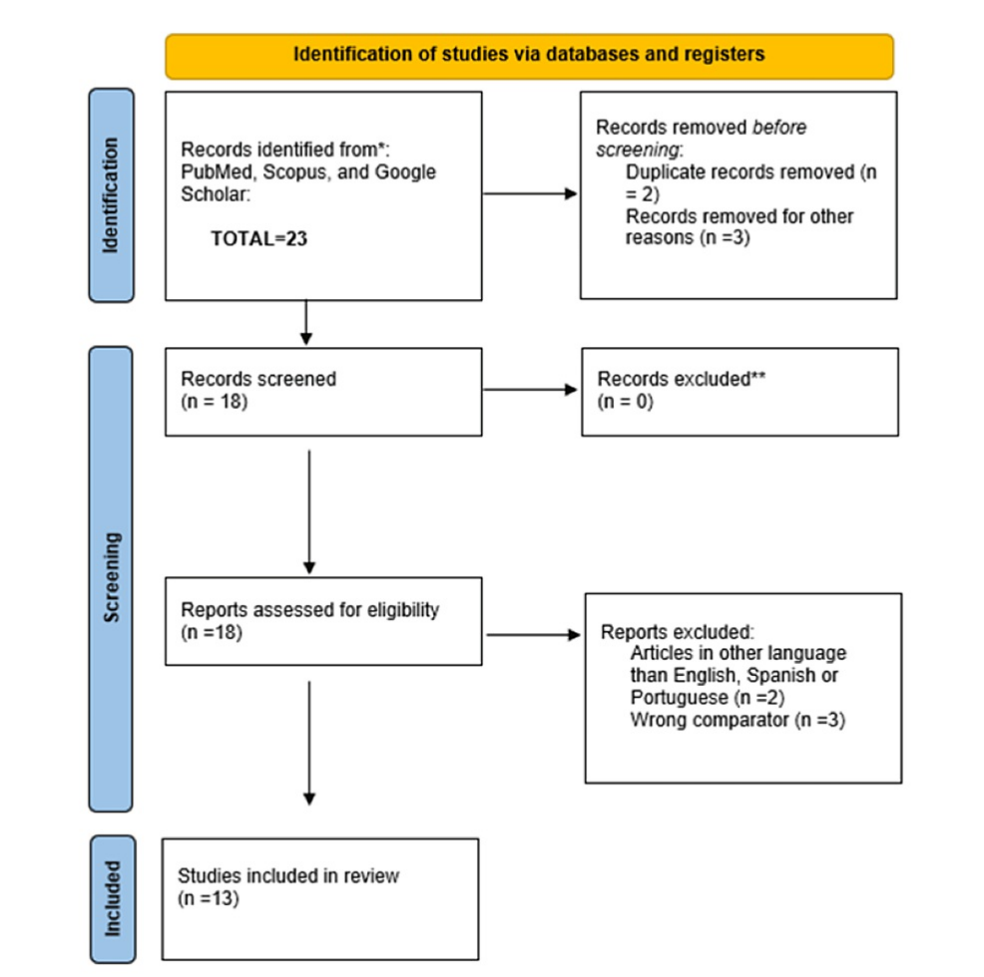
Flowchart of the studies

For this narrative review, an exploration of the existing literature was undertaken to identify articles pertinent to artifacts in the vHIT. Databases such as PubMed and Scopus were reviewed, using keywords such as "vHIT artifacts," "vestibulo-ocular reflex," and "diagnostic challenges." Articles in English, Portuguese, and Spanish published up to the specified cutoff date of 2017 to 2024 were considered for inclusion as seen in Figure 1.

The inclusion criteria were tailored to encompass articles focusing on the discussion of artifacts associated with the vHIT. Studies that delved into other vestibular assessments such as posturography, caloric testing, vestibular evoked myogenic potentials, dynamic visual acuity test, and rotary chair test or unrelated topics were deliberately excluded to maintain thematic relevance. Both experimental and clinical studies were considered, ensuring a diverse representation across various populations and settings.

The data extraction process involved distilling pertinent information from selected articles, including study design, participant demographics, vHIT protocols, identified artifacts, and proposed solutions.

Our approach to data synthesis involved a qualitative analysis of the identified artifacts, emphasizing a comprehensive understanding of their implications for vHIT outcomes. Additionally, emerging methodologies and strategies proposed in the literature to mitigate the impact of artifacts were synthesized, offering a nuanced perspective on the current state of knowledge.

Review

The vHIT has become a cornerstone in the assessment of vestibular function, offering valuable insights into the integrity of the VOR [1,3,4]. In the comprehensive exploration of artifacts associated with the vHIT, a crucial step involved the systematic categorization of identified artifacts into four primary groups: technological, patient-related, environmental factors, and evaluator-related factors [1,3,4]. Technological artifacts specifically addressed limitations inherent in eye-tracking systems and software, dissecting the intricacies that contribute to variations in vHIT outcomes [4,7]. Patient-related factors encompassed considerations such as age, comorbidities, and the level of patient cooperation, shedding light on the diverse individual variables that can impact the fidelity of vHIT results [8]. Furthermore, the review scrutinized environmental factors, delving into nuanced aspects such as ambient lighting conditions and calibration issues [4,7]. Evaluator-related factors significantly influence the precision and reliability of the vHIT, encompassing expertise, communication, technique, and data interpretation [1,8]. This categorization framework provides a structured lens through which to analyze the multifaceted challenges encountered in vHIT applications, offering insights into the intricate interplay between technology, patient characteristics, environmental conditions, and evaluator-related factors within the context of vestibular diagnostics [1,4]. 

Identifying artifacts in vHIT

Accurate interpretation of the vHIT relies heavily on the examiner’s ability to discern genuine vestibular pathology from artifacts [9,10]. A thorough understanding of the potential sources of artifacts is paramount in ensuring the reliability of diagnostic outcomes [9,11]. Among the multifaceted challenges, technological artifacts often surface as calibration errors, gaze deviations, or delays in eye movement recordings [9,11]. Patient-related factors, including variations in head movement dynamics, inconsistent cooperation levels, and the influence of comorbidities on eye movement patterns, further complicate the interpretation of vHIT results [9,11]. Additionally, environmental artifacts, such as ambient lighting fluctuations or suboptimal testing conditions, can introduce confounding elements into the diagnostic process [9,11]. Evaluator-related factors significantly influence the precision and reliability of the vHIT, encompassing expertise, communication, technique, and data interpretation [1,8].

Type of artifacts

Technological Artifacts 

The technological underpinnings of vHIT, particularly eye-tracking systems and software, introduce a host of potential artifacts [9]. Enumerating these technological artifacts:

Eye-tracking system limitations: Inaccuracies or limitations in the eye-tracking system, such as reduced sampling rate or tracking errors, can contribute to artifacts in vHIT recordings [9].

Software limitations: Issues related to the software used for data analysis and interpretation, including algorithms for artifact correction and gaze stabilization, may introduce inaccuracies in vHIT outcomes [9].

Calibration errors: Incomplete or inaccurate calibration of the system, including the head and eye-tracking components, can result in artifacts during the test, affecting the precision of recorded eye movements [9].

Technological lag: Delay or lag in the responsiveness of the equipment may introduce discrepancies between the actual head movements and the recorded eye movements, leading to artifacts [9].

Interference and environmental factors: External factors, such as ambient lighting, electromagnetic interference, or calibration issues in the testing environment, can affect the performance of the technological components and contribute to artifacts [9]. The best illumination for a vHIT performance is typically achieved with bright, uniform, and even lighting in the testing area. This ensures clear visibility of the patient's eyes and accurate recording of eye movements, minimizing potential errors and leading to reliable test results [9]. Additionally, it is important to avoid harsh shadows and glare on the patient's face and eyes to maintain optimal test conditions [12].

Equipment malfunction: Mechanical or electronic malfunction of the vHIT equipment, including issues with the head impulse device including the eye-tracking goggles and elastic headband, can introduce artifacts in the recorded data [9].

Patient-Related Challenges

Beyond technological considerations, vHIT outcomes are intricately linked to patient-specific factors [10]. Age-related changes in vestibular function, variations in cooperation levels, eye-opening, and the presence of comorbidities can introduce variability in test results [10]. The challenge lies in discerning whether deviations from expected outcomes are indicative of true vestibular dysfunction or attributable to these patient-related factors [10]. Some of them are listed below.

Cooperation level: Patient cooperation is crucial for the accurate performance of the vHIT. Limited cooperation, anxiety, overacted collaboration, or discomfort may impact the patient's ability to follow instructions and execute precise head movements during the test [10,11].

Age-related factors: The vestibular system can be influenced by age, particularly in pediatric and older patients, where changes in vestibular function may be observed. It is crucial to comprehend and consider age-related variations to interpret vHIT results accurately [10,11].

Comorbidities: Patients with underlying medical conditions, especially those affecting the vestibular system or eye movements, may present challenges in obtaining clear and reliable vHIT recordings. Coexisting health issues can complicate the interpretation of test outcomes. [10,11]. Other cervical and neck conditions may complicate the performance of the vHIT, such as cervical spondylosis, cervical arthrosis, cervical radiculopathy, whiplash injury, recent neck surgery, rheumatoid arthritis, and severe neck pain [10,11].

Medication effects: Certain medications may affect vestibular function or eye movements, potentially influencing vHIT results. Awareness of the patient’s medication history is crucial for accurate interpretation and diagnosis [10,11].

Cognitive impairment: Patients with cognitive impairments or neurological disorders may face challenges in understanding and following the instructions during the vHIT, affecting the overall reliability of the test [10,11].

Pregnancy and hormonal influences: In specific contexts, such as postmenopausal women, hormonal fluctuations may influence vestibular function, adding complexity to the interpretation of vHIT outcomes [10,11]. In pregnant females, increased gains have been described mainly in the lateral canals [13].

Environmental Influences

The environment in which the vHIT is conducted can also contribute to artifacts [9]. Ambient lighting conditions, for example, may interfere with accurate eye-tracking, affecting the precision of VOR measurements [9]. The following are considered some of the most widely known environmental influences on artifacts:

Ambient lighting: Inadequate or inconsistent ambient lighting in the testing environment can impact the accuracy of the vHIT by affecting the visibility of the patient's eyes and compromising the tracking system [9-11].

Calibration issues: Environmental factors, such as changes in temperature or humidity, may contribute to calibration issues in the equipment, potentially leading to inaccuracies in the recorded eye movements [9-11].

Noise and distractions: External noise or distractions in the testing environment can disrupt the concentration of both the patient and the examiner, potentially influencing the patient's ability to execute precise head movements [9-11].

Space constraints: Limited space in the testing area may restrict the range of head movements that can be comfortably performed by the patient, affecting the completeness and accuracy of vHIT recordings [9-11].

Electromagnetic interference: The presence of electronic devices or electromagnetic interference in the vicinity of the testing equipment may interfere with the proper functioning of the vHIT technology, leading to artifacts in the data [9-11]. To minimize electromagnetic interference during the vHIT, it is crucial to have a dedicated testing room that is free from electronic devices emitting electromagnetic radiation [9-11], ensure proper electromagnetic shielding of the vHIT equipment, use shielded cables to reduce interference, and additionally, keep the room free from metallic objects and regularly maintain and check the vHIT equipment to ensure proper function [9-11].

Temperature fluctuations: Extreme temperature variations in the testing environment may impact the performance of the equipment and affect the patient's comfort during the vHIT, potentially influencing test outcomes [9-11].

Evaluator-Related Factors

The performance of the vHIT relies not only on the equipment and patient cooperation but also on the expertise and actions of the evaluator. Here are some important evaluator-related factors that can influence the accuracy and effectiveness of the vHIT [1,9-11].

Experience and training: The evaluator should have the necessary training and experience in conducting the vHIT. Familiarity with the equipment, testing protocols, and interpretation of results is crucial for accurate testing [1,9-11].

Patient communication: Effective communication with the patient is essential. The evaluator should explain the test procedure, ensure the patient understands their role, and address any concerns or questions the patient may have [1,9-11].

Patient preparation: Properly preparing the patient for the test is important. This includes explaining the importance of maintaining a stable gaze on a target during head impulses and ensuring the patient is comfortable with the testing equipment, including the head-mounted goggles or glasses [1,9-11].

Gaze stabilization: The evaluator should monitor the patient's gaze stabilization throughout the test. This involves observing whether the patient can maintain fixation on a visual target during rapid head movements [1,9-11]. A double-check of gaze stabilization is suggested by the evaluator or his/her team [1,9-11].

Head impulse technique: The evaluator's technique in delivering head impulses should be consistent and controlled. This includes the amplitude and timing of head movements to stimulate the vestibular system effectively [1,9-11].

Challenges in artifact identification

Distinguishing between authentic vestibular dysfunction and artifacts poses a formidable challenge [4,10]. The inherent variability in vHIT outcomes, coupled with the dynamic nature of head movements during testing, necessitates a nuanced approach to recognize and address potential artifacts [4,10]. The interplay of technological, patient-related, and environmental factors requires a comprehensive strategy to accurately identify and mitigate potential confounders in vHIT recordings [4,10].

Technological Challenges and Solutions

Calibration inaccuracies, a recurring technological artifact, demand meticulous calibration procedures and regular system checks [4,10]. The integration of advanced eye-tracking technologies with higher sampling rates and improved signal processing capabilities represents a pivotal step in reducing misinterpretations resulting from technological limitations [4,10]. The continuous evolution of hardware and software promises enhanced precision and reliability in vHIT recordings, offering encouraging avenues to overcome technological challenges [4,10].

Patient-Related Considerations

Effectively addressing patient-related challenges involves tailoring vHIT protocols to various demographic groups [10]. Customizing testing procedures based on age groups, optimizing instructions to enhance patient cooperation, and accounting for the influence of comorbidities on eye movement dynamics contribute to minimizing patient-related artifacts [10]. The development of standardized procedures to assess and account for individual patient factors ensures a more accurate interpretation of vHIT results across diverse clinical scenarios [10].

Environmental Influences and Standardization

The impact of environmental artifacts, such as changes in ambient lighting, underscores the necessity for standardized testing conditions [10,12]. Implementing guidelines for optimal testing environments serves to mitigate external factors' impact on vHIT outcomes [10,12]. Shielding the testing area from excessive light variations and maintaining consistent conditions across different testing sessions contribute to a more controlled and reproducible testing environment [10,12]. 

Avoiding artifacts

Artifacts in the vHIT, arising from technological, patient-related, and environmental factors, possess the capability to distort test results and compromise the validity of vestibular assessments [4]. The implementation of advanced calibration techniques, such as multi-point calibrations and real-time adjustments, significantly enhances the accuracy of vHIT recordings and effectively mitigates calibration-related artifacts [14]. Additionally, the integration of adaptive algorithms that dynamically adjust for artifacts in real time emerges as a promising approach to bolster the robustness of vHIT data interpretation [14]. These adaptive algorithms play a crucial role in compensating for unexpected variations during testing, contributing to more reliable and accurate outcomes [14]. Moreover, the establishment and adherence to standardized vHIT protocols, incorporating age-specific guidelines and recommendations for addressing patient-related factors, ensure consistency in testing procedures across diverse populations [12,13]. This standardization promotes uniformity in both data acquisition and interpretation [12,13]. To further elevate proficiency in vHIT administration, continuous education and training for clinicians are imperative [12,14]. Staying informed about technological advancements and refining skills enable practitioners to adeptly navigate the nuances associated with the vHIT in real-world clinical settings, ultimately improving the overall efficacy of vestibular diagnostics [12,14].

Controversies in vHIT artifacts

While the vHIT has gained prominence as a valuable tool for vestibular assessment, it is not without its share of controversies [15,16]. These controversies highlight the ongoing debates and uncertainties surrounding the application and interpretation of the vHIT in clinical practice [16-18]. Controversies in the interpretation of normative data for the test constitute a contentious realm, marked by variations in defining "normal" VOR gain values across studies [18-20]. The absence of standardized normative values raises concerns about the generalizability and comparability of vHIT results across diverse populations [20,21]. The influence of age on vHIT outcomes is another area of debate, with challenges in establishing clear age-specific criteria for abnormal results [22]. Disagreements persist regarding whether adjustments for age-related variations should be incorporated into diagnostic thresholds, impacting the sensitivity and specificity of the vHIT [22]. The ongoing debate surrounding artifact recognition and correction in vHIT recordings underscores the need for standardized procedures to enhance reliability and reproducibility [23]. Additionally, determining the clinical relevance of small VOR gain changes remains a source of debate, with implications for treatment decisions [24]. The lack of standardized protocols for vHIT administration further contributes to controversies, challenging the comparability of results across clinics and studies [24]. Standardizing protocols is an ongoing effort to enhance the reproducibility and reliability of vHIT outcomes [24]. 

While the existing literature on artifacts in the vHIT provides valuable insights into various challenges and mitigation strategies, it is essential to acknowledge certain limitations inherent in this body of work. The current literature exhibits heterogeneity in methodologies, with differing approaches to artifact identification and correction across studies. Variations in sample sizes, participant demographics, and vHIT protocols further contribute to the complexity of synthesizing findings [10-24]. Additionally, a notable portion of the literature lacks standardized criteria for defining and categorizing artifacts, leading to potential ambiguity in the interpretation of results [10-24]. The paucity of longitudinal studies and limited exploration of certain demographic groups also present gaps in our understanding of the persistence and impact of artifacts over time [10-24]. Recognizing these limitations is crucial for fostering a nuanced appreciation of the existing literature and underscores the need for future research efforts aimed at establishing more standardized methodologies and addressing specific gaps in knowledge [10-24]. 

Artifacts frequently documented in academic works and future perspectives

vHIT interpretation can be complicated by several artifacts, such as slippage, calibration discrepancies, blinks, multiple VOR peaks, trace oscillations, incorrect VOR direction, saccades organization and classification, and unclassifiable artifacts [17]. It is imperative to comprehend and address these artifacts to guarantee dependable vHIT outcomes, thereby enhancing diagnostic precision in individuals with vestibular dysfunction [17]. Among the artifacts commonly reported in the literature are the following:

Slippage

There might be displacement of the head-to-eye movement during the vHIT [17]. This can occur when the goggles are not securely fastened, causing abnormal reading of the eye movements as it would cause dissociation between the head and eye movements [17]. Other potential causes of slippage include (1) unintentional contact with the goggles during testing and (2) too tight of a headband, resulting in stretching of the patient's skin [17]. Slippage can cause abnormal VOR gain values without apparent vestibular deficits or can mask deficits by producing normal gain values alongside re-fixation saccades [17].

Calibration Problems

These occur when participants fail to follow instructions accurately. Signs of inadequate calibration include VOR gain values that are excessively high (>1.3) or low (<0.79) without subsequent re-fixation saccades [17].

Blinks During vHIT Testing

These are characterized as eye movements that cross the baseline, with peak eye velocity comprising at least 75% of the blink [17].

Multiple VOR Peaks

Multiple VOR peaks were defined as two or more eye velocity peaks during head movement, with each peak representing at least 25% of the peak eye velocity [17]. This can complicate accurate gain calculation and may result from various factors such as environmental interference, mini-blinks, or issues with pupil detection.

Trace Oscillations

These are small eye movements that occur during or after head movement and are classified as refixation saccades. These movements typically involve less than 25% of the peak eye velocity during head movement and less than 75% of peak eye velocity afterward, hindering the assessment of saccades and gain calculation [17].

Wrong VOR Direction

This occurs when there is either no eye movement or eye movement in the same direction as head movement, often due to patient inattention [17].

Unclassifiable Artifacts

Unclassifiable artifacts are any other disturbances that impede interpretation but do not fit the criteria outlined above. These artifacts may present challenges in data analysis and interpretation during the vHIT [17].

As technology continues to advance, the future of artifacts in the vHIT holds promise for both refinement and mitigation [15]. With ongoing research and development, innovative solutions are anticipated to emerge to address common artifacts encountered in vHIT recordings [15]. Improved sensor technologies, enhanced signal processing algorithms, and advancements in calibration methods are expected to continuously improve test accuracy and minimize artifact occurrence [25].

Moreover, interdisciplinary collaborations between engineers, neuroscientists, and clinicians are likely to lead to novel approaches for artifact identification and correction, ultimately enhancing the reliability and clinical utility of vHIT as a diagnostic tool for vestibular disorders [6]. As virtual reality and augmented reality technologies continue to evolve, there is potential for the integration of immersive visualization techniques into vHIT systems, facilitating enhanced training for clinicians and offering new insights into vestibular function [26]. Overall, the outlook for artifacts in vHIT is one of ongoing refinement and innovation, intending to continually improve diagnostic accuracy and patient care in the field of vestibular medicine [15].

Future studies could encompass various avenues. Establishing standardized criteria for artifact identification and categorization is pivotal, aiming to foster consensus guidelines that enhance result comparability across different studies and clinical contexts. Longitudinal assessments could provide valuable insights into the persistence and impact of artifacts over time, particularly in various phases of vestibular disorders. Population-specific considerations, including age-related factors and comorbidities, warrant exploration to tailor normative data and improve the precision of vHIT diagnostics for diverse groups. Comprehensive comparison studies with other vestibular assessment modalities would shed light on the concordance and diagnostic accuracy of the vHIT, refining its role in comprehensive vestibular evaluations. Investigating the clinical impact of small changes in VOR gain is crucial for treatment decision-making, requiring studies to establish clinically meaningful thresholds. Additionally, exploring advanced technological solutions, such as improved eye-tracking systems and the integration of artificial intelligence, could contribute to real-time artifact recognition and correction, refining the overall accuracy of vHIT outcomes. These future research directions aim to address current gaps in knowledge and enhance the reliability of the vHIT as a diagnostic tool.

## Conclusions

In summary, this review has delved into the complexities of artifacts in the vHIT, providing insights into challenges, controversies, and mitigation strategies. Categorizing artifacts, exploring advanced techniques, and addressing controversies highlighted the dynamic nature of vestibular diagnostics. While acknowledging limitations in the current literature, this review calls for standardized methodologies and continued research to advance the accuracy and reliability of the vHIT. As we navigate these challenges, our collective efforts will undoubtedly contribute to refining diagnostic practices and elevating patient care in vestibular medicine.
